# Investigation on the Regional Loss Factor and Its Anisotropy for Aortic Aneurysms

**DOI:** 10.3390/ma9110867

**Published:** 2016-10-26

**Authors:** Nastaran Shahmansouri, Mohammed Alreshidan, Alexander Emmott, Kevin Lachapelle, Ismaïl El-Hamamsy, Raymond Cartier, Richard L. Leask, Rosaire Mongrain

**Affiliations:** 1 Department of Mechanical Engineering, McGill University, Montreal, QC H3A 0C3, Canada; nastaran.shahmansouri@mail.mcgill.ca; 2 Research Center, Montreal Heart Institute, Montreal, QC H1T 1C8, Canada; alexander.emmott@mail.mcgill.ca (A.E.); ielhamamsy@yahoo.ca (I.E.-H.); RC2910@aol.com (R.C.); richard.leask@mcgill.ca (R.L.L.); 3 Division of Cardiac Surgery, Royal Victoria Hospital, Montreal, Montreal, QC H4A 3J1, Canada; mohammed.alreshidan@mail.mcgill.ca (M.A.); kevin.lachapelle@mcgill.ca (K.L.); 4 Department of Chemical Engineering, McGill University, Montreal, QC H3A 2B2, Canada; 5 Division of Cardiac Surgery, Montreal Heart Institute, Université de Montréal, Montreal, QC H1T 1C8, Canada

**Keywords:** aortic aneurysm, loss factor, strip-biaxial tensile test, age, aortic diameter, collagen and elastin

## Abstract

An aortic aneurysm is a lethal arterial disease that mainly occurs in the thoracic and abdominal regions of the aorta. Thoracic aortic aneurysms are prevalent in the root/ascending parts of the aorta and can lead to aortic rupture resulting in the sudden death of patients. Understanding the biomechanical and histopathological changes associated with ascending thoracic aortic aneurysms (ATAAs), this study investigates the mechanical properties of the aorta during strip-biaxial tensile cycles. The loss factor—defined as the ratio of dissipated energy to the energy absorbed during a tensile cycle—the incremental modulus, and their anisotropy indexes were compared with the media fiber compositions for aneurysmal (*n* = 26) and control (*n* = 4) human ascending aortas. The aneurysmal aortas were categorized into the aortas with bicuspid aortic valves (BAV) and tricuspid aortic valves (TAV). The strip-biaxial loss factor correlates well with the diameter of the aortas with BAV and TAV (for the axial direction, respectively, *R*^2^ = 0.71, *p* = 0.0022 and *R*^2^ = 0.54, *p* = 0.0096). The loss factor increases significantly with patients’ age in the BAV group (for the axial direction: *R*^2^ = 0.45, *p* = 0.0164). The loss factor is isotropic for all TAV quadrants, whereas it is on average only isotropic in the anterior and outer curvature regions of the BAV group. The results suggest that loss factor may be a useful surrogate measure to describe the histopathology of aneurysmal tissue and to demonstrate the differences between ATAAs with the BAV and TAV.

## 1. Introduction

Elective cardiac surgery is used to prevent the rupture and complications of ascending thoracic aortic aneurysms (ATAAs). While size is the main clinical measure for aortic replacement surgery, there are uncertainties about the efficiency of this measure [1,2,3]. It is widely recognized that diameter alone is only modestly appropriate for assessing ATAA risk and function [3,4]. To better guide surgical decision making, a biomechanical surrogate measure estimating ATAA wall properties is needed to better stratify patients.

A large number of investigations on the biomechanics of ascending thoracic aortas (ATAs) and ATAAs have addressed the incremental modulus [5,6,7] and ultimate tensile strength [7,8,9] of the aortas. Both of these mechanical properties provide information on a certain stress or strain level of the tensile curve. Although it is possible to estimate the incremental modulus non-invasively from in vivo data using medical imaging [10], the ultimate tensile strength has the shortcoming of being unmeasurable in vivo. Furthermore, the ATA tissue under physiological condition normally does not reach the level of ultimate tensile strength which shows another limitation of this property for a global characterization of ATAs [5,11].

Strain energy functions have been used for detailed biomechanical characterization of the ATA and ATAA walls [6,7,9,11,12,13,14]. Energy functions have the advantage of providing information on the entire tensile stress and strain ranges. Seeing the benefits of energy approaches to study the tissue biomechanics, the energy loss of the tissue during cyclical loading has been used to link ATAA medial degeneration with its biomechanical function [15]. The energy loss surrogate measure has been proposed by Chung et al. to better represent functional and histologic changes with aneurysms [15]. The loss factor (energy loss) is a dimensionless parameter representative of tissue absorbed and dissipated energy levels during loading and unloading tensile cycles with the aforementioned advantage of strain energy functions. Whereas the energy functions could give the opportunity of detailed structural analysis of the aortic wall in different regions and across its thickness, the loss factor introduces a tangible surrogate measure, which may be estimated noninvasively using medical imaging techniques. This parameter also provides information on Windkessel function of the aorta because it is a measure of energy dissipated by the aortic wall subjected to a tensile cycle [16]. 

The mechanical properties of the ATA wall show directional dependency [17,18,19,20] and that it is inhomogeneous with respect to region and the aortic layers [8,13,17,19]. The mechanical response of the ATA is governed by the properties of its layers, the intima, media, and adventitia, which could be associated with non-uniform tissue remodelling during the progression of aortic aneurysms [21,22]. It is reported that the media of ATAAs are significantly thicker than the adventitia (three to four times) and intima (five to six times) [23]. Aneurysms are classically attributed to media degeneration which is most often a non-inflammatory loss of smooth muscle cells and fragmentation of elastic fibers [24,25]. The anisotropy index of mechanical data is likely to change with medial degeneration, becoming more amorphous and isotropic as the ATAA progresses.

In this study, we explored the apparent (incremental) modulus, loss factor and anisotropy indexes in ATA and ATAA samples, categorized into bicuspid aortic valve (BAV) and tricuspid aortic valve (TAV) groups. We compared these results with the media fiber compositions. The biomechanical properties of the control ATAs (*n* = 4), the BAV (*n* = 12), and the TAV (*n* = 14) ATAAs were evaluated using planar strip-biaxial tensile tests in the axial and circumferential directions. The loss factor and incremental modulus of the tissue, as well as, the anisotropy of these mechanical properties were determined and compared regionally amongst the aortic quadrants. The comparisons of these properties with collagen and elastin contents, aortic diameter, and patients’ age have been scrutinized. 

## 2. Materials and Methods

### 2.1. Tissue Collection and Preparation

Aneurysmal (*n* = 26) and control (*n* = 4) ATAs were collected and examined with informed consent and ethical approval at the Montreal Heart Institute and McGill University Health Network. The aneurysmal aortic tissues were obtained during surgery, and the control aortas were collected from heart transplant recipients and patients undergoing the Ross procedure with normally sized aortas. The aortas were kept refrigerated in a buffer solution after the tissue collection and were examined within 24 h. The characteristics of the patient population, including the ATAAs with BAV (*n* = 12) and the TAV (*n* = 14), are reported in Table 1. 

**Figure 1:**
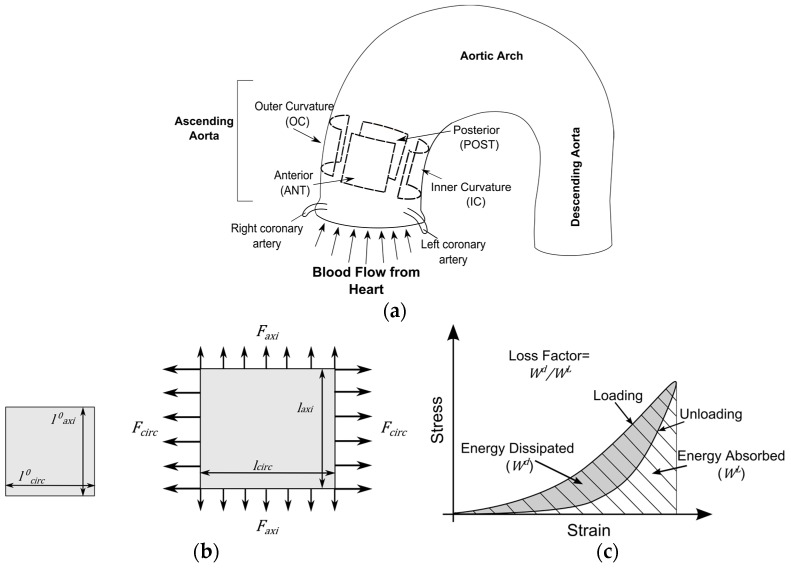
(**a**) Illustration of the ascending thoracic aorta (ATA) and the aortic quadrants; (**b**) A tissue sample in its initial load-free configuration (**left**), and when it is under the biaxial tensile loads (**right**); (**c**) Typical non-linear stress-strain behavior of a tissue sample subjected to a loading-unloading tensile cycle and the definition of loss factor.

The circumference of the aortas was measured and used to approximate their ex vivo diameter. The tests were performed on tissue sections with the size of 15 × 15 mm^2^ excised from four quadrants of the aortic rings: Anterior (Ant), Outer Curvature (OC), Posterior (Post), and Inner Curvature (IC), see Figure 1a. The thickness of the sections measured at five different points using a constant force digital thickness caliper (Mitutoyo: Litematic VL-50A, Mitutoyo Corp., Kanagawa, Japan) and the average value was used in the analysis.

### 2.2. Biaxial Tensile Tests

#### 2.2.1. Method of Experiments

Biaxial tensile tests were performed utilizing an EnduraTec ELF 3200 (Bose Corporation, Eden Prarie, MN, USA), which has two perpendicular axes equipped with a displacement transducer and a 1 kg load-cell (Model 31, Honeywell Sensotec, Morris Plains, NJ, USA). Four silk surgical threads model 4–0 (Ethicon, Somerville, NJ, USA) with adjusted suture hooks were attached to the four edges of the tissue patches. These threads were used to affix the samples to the tensile tester grips. The relaxed dimensions between the sutures were determined by a caliper at two locations in both the circumferential and the axial directions; the average values were used in the subsequent analysis. To avoid tissue shear stresses in the samples, careful considerations were taken to make their edges parallel to the axes of the mechanical tester. The samples were preloaded in both directions by 0.050 N to ensure the sutures are a little tensed and the tissues were in the same stress condition in both directions. Afterwards, the samples were subjected to 13 equi-biaxial preconditioning cycles by stretching the sutures in both the circumferential and axial direction by 6 mm. 

The preconditioning cycles were followed by three strip-biaxial experimental cycles in either the circumferential and axial directions. During the experiments, one axis was kept immobile, whereas the other axis was stretched by 6 mm at a rate of 0.1 mm/s to obtain a quasi-static state for the tensile tests. The displacements of the sutures in both directions were controlled by Wintest software (Bose Corporation, Eden Prarie, MN, USA) which also displayed and recorded the load and displacement data. The load and displacement data of three experimental cycles was averaged and used in the analysis. Throughout the experiments, the specimens were submerged in a bath of buffered saline solution at 37 °C temperature. Figure 1 illustrates a specimen in the initial load-free configuration and when it is subjected to strip-biaxial loads. 

The collagen and elastin contents for the aortic media were estimated by histologic image analysis of formalin fixed Movat’s Pentachrome stained sections using a similar method explained in our previous work [26]. Three images covering roughly 1/3rd of the media were captured by a Leitz Diaplan microscope and using an Infinity 1 (Lumenera Corp., Ottawa, ON, Canada) Camera utilizing a 10× object lens and processed by ImageJ color detection. The histological analysis was performed blinded to the mechanical data.

#### 2.2.2. Method of Analysis

The second Piola-Kirchhoff stress and Green strain definitions are a work-conjugate stress-strain pair used in this work to obtain the strip biaxial tensile behavior of the aortas and to calculate their loss factors.

The Green strains are defined as

(1) $$E_{circ}=\frac{{\left(\lambda_{circ}\right)}^{2}-1}{2}\;\mathrm{and}\;E_{axi}=\frac{{\left(\lambda_{axi}\right)}^{2}-1}{2}$$

where the stretches, $\lambda_{circ}$ and $\lambda_{axi}$, are given by

(2) $$\lambda_{circ}=1+dl_{circ}/l_{circ}^{0}\;\mathrm{and}\;\lambda_{axi}=1+dl_{axi}\;/l_{axi}^{0}$$

in which $dl_{circ}$ and $dl_{axi}$ are the displacements of sutures, and $l_{circ}^{0}$ and $l_{axi}^{0}$ are the initial distances between the sutures respectively in the circumferential and axial directions. 

The second Piola-Kirchhoff stresses in the circumferential and axial directions are defined as

(3) $$S_{circ}=\frac{F_{circ}}{A_{circ}^{0}\lambda_{circ}}=\frac{F_{circ}}{A_{circ}^{0}\sqrt{2E_{circ1}+1}},\;\mathrm{and}\;S_{axi}=\frac{F_{axi}}{A_{axi}^{0}\lambda_{axi}}=\frac{F_{axi}}{A_{axi}^{0}\sqrt{2E_{axi}+1}},$$

in which, $A_{circ}^{0}$ and $A_{axi}^{0}$ are the initial areas, and $F_{circ}$ and $F_{axi}$ are the loads in circumferential and axial directions, correspondingly. In the calculations, the load and strain values were assumed to be 0 at the start of the experimental cycles. The stretches were 1 at the beginning of these cycles ($E_{i0}=0,\;i=circ,axi$). The thickness and the lengths between the sutures measured at initial load-free configuration were used to determine $A_{circ}^{0}$ and $A_{axi}^{0}$.

The loss factor is the ratio of energy dissipated during the loading (*L*)–unloading (*U*) cycle ($W^{d}$) and the energy absorbed during the loading phase ($W^{L}$) (see Figure 1). Hence, the loss factor is

(4) WiL=∫0max(Ei)SiL(EiL)dEiL,Wid=WiL−WiU=∫0max(Ei)SiL(EiL)dEiL−∫0max(Ei)SiU(EiU)dEiU,LFi=WidWiL, and i=circ, axi

In this study, considering the controlling system of the device and the presented data in the literature, the location of sutures were used to obtain the tensile properties and loss factors of the aortas. 

The stress and strain data of the loading tensile curves were used to find incremental modulus at a strain value of 30%. To do this, the slope of first-order polynomials fitted to the stress-strain data at the strain range of 30% ± 2% were evaluated. 

The anisotropy of the mechanical properties is defined as

(5) $$MP\;anisotropy=\frac{MP_{axial}-MP_{circ}}{(MP_{axial}+MP_{circ})/2}$$

where *MP* is the loss factor or the incremental modulus and $MP_{axial}$ and $MP_{circ}$ are the corresponding values in the axial and circumferential directions, respectively. 

### 2.3. Statistical Analyses

Statistical analysis was performed on data obtained using Prism V5.0 software (GraphPad Software Inc., San Diego, CA, USA). One-way analysis of variance (ANOVA) were conducted to examine the effect of tissue groups and aortic quadrants; we assumed that samples of the BAV and TAV groups follow normal distributions. Bonferroni’s multiple comparisons post-test was executed in addition to ANOVA analysis. For one-way ANOVA tests, the mean values and the standard deviations are reported to compare the data between the groups or regions. Analysis including control group—comparisons of three tissue groups—was done by nonparametric ANOVA (Kruskal-Wallis analysis) and the median and interquartile are presented in the corresponding images.

The results were considered to be significantly different with *p*-values ≤ 0.05. Linear regressions were employed to investigate the correlation between different parameters and properties. The correlations were considered significant when the slope of the linear regression was significantly different from zero and the *p*-value ≤ 0.050. 

## 3. Results

The loss factor and incremental modulus were evaluated for both the axial and circumferential directions of the aortic quadrants and the anisotropic characteristics of these mechanical properties were determined. Additionally, the contents of structural fibers for the media were measured. From data obtained, the variation of the average mechanical properties and the fiber contents were investigated amongst the tissue groups and the aortic regions. The mechanical characteristics were also compared with the collagen and elastin content, aortic diameter, and patients’ age. 

### 3.1. Loss Factor and Loss Factor Anisotropy

**Figure 2:**
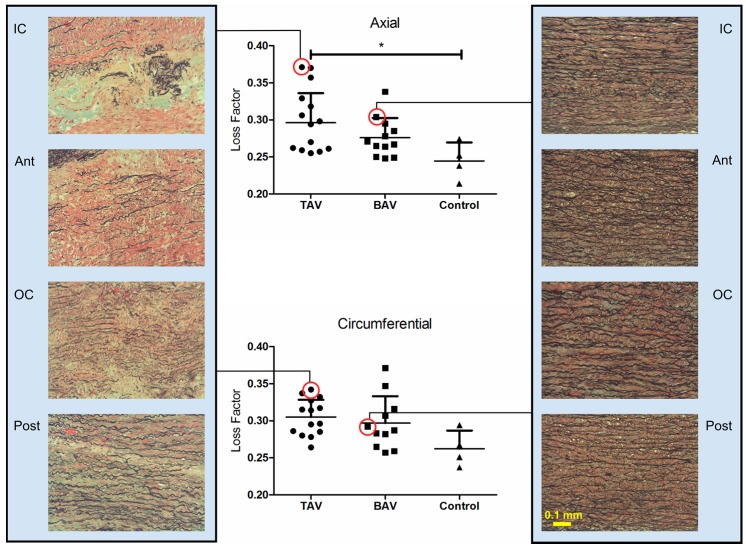
Average loss factor for the three tissue groups in the axial and circumferential directions. Average axial loss factor depends on groups (*p* = 0.0419) (Kruskal-Wallis and Dunn’s multiple comparisons post-test). Asterisk shows significant difference: (*) *p* < 0.05. The figure also presents the histology images for the aortic quadrants of a 76-year-old patient from the TAV group and an 81-year-old patient from the BAV group.

The average axial and circumferential loss factor of the aortic quadrants of the three tissue groups are compared in Figure 2. The analysis indicated that the median axial loss factor was dependent on the tissue group (*p* = 0.0419, Kruskal-Wallis), and that the loss factor of the TAV group was significantly higher than the control group in the axial direction (*p* < 0.05, Dunn’s multiple comparison test). It should be noted that the difference in the number of BAV samples in the circumferential (*n* = 11) and axial (*n* = 12) directions is due to omitting the circumferential data of one BAV sample because of the presence of experimental noise. 

**Figure 3:**
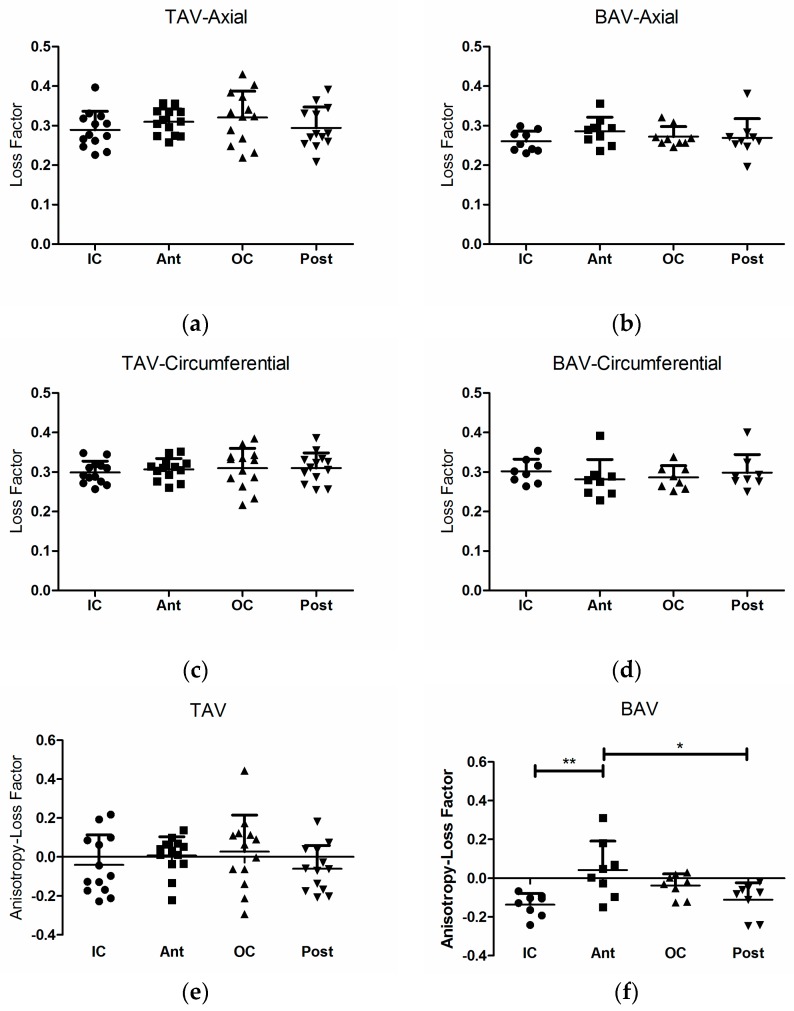
Variation of axial loss factor: (**a**) TAV; and (**b**) BAV groups. Variation of circumferential Loss Factor: (**c**) TAV group; and (**d**) BAV group; Variation of loss factor Anisotropy: (**e**) TAV: No regional effect; (**f**) BAV: significant regional effect (*p* = 0.0021) (one-way analysis of variance (ANOVA) with repeated measure and Bonferroni’s multiple comparisons post-test). Asterisks denote significant difference: * *p* < 0.05 and ** *p* < 0.01.

Figure 3 presents the regional variations of the loss factor for the axial and circumferential directions of TAV groups (*n* = 13) and the BAV group (*n* = 9 and 8 for the axial and circumferential directions). The analysis was performed using one-way ANOVA statistical method with repeated measures, which require that only the aortas with available data for all quadrants be included in the analysis. The loss factor did not show regional variation in the axial or the circumferential direction for these groups. The regional variation of loss factor anisotropy for the BAV and TAV groups are also shown in Figure 3e,f. The loss factor anisotropy did not vary amongst the three tissue groups (one-way ANOVA, *p* = 0.3889). The loss factor anisotropy of the four aortic quadrants of the TAV group did not vary significantly from zero, hence, all TAV quadrants could be considered isotropic (one-sample *t*-test). The inner curvature and posterior region of the BAV tissue were anisotropic (one-sample *t*-test, *p* (two-tailed) = 0.0003 and 0.0090 for the IC and Post regions, respectively). The BAV group also showed significant regional variation in loss factor anisotropy (one-way ANOVA with repeated measure, *p* = 0.0021). The anterior region had the highest anisotropy (loss factor) which was significantly greater than the inner curvature and posterior regions (Bonferroni’s multiple comparisons post-test, *p* < 0.01 and *p* < 0.05, respectively).

**Figure A1:**
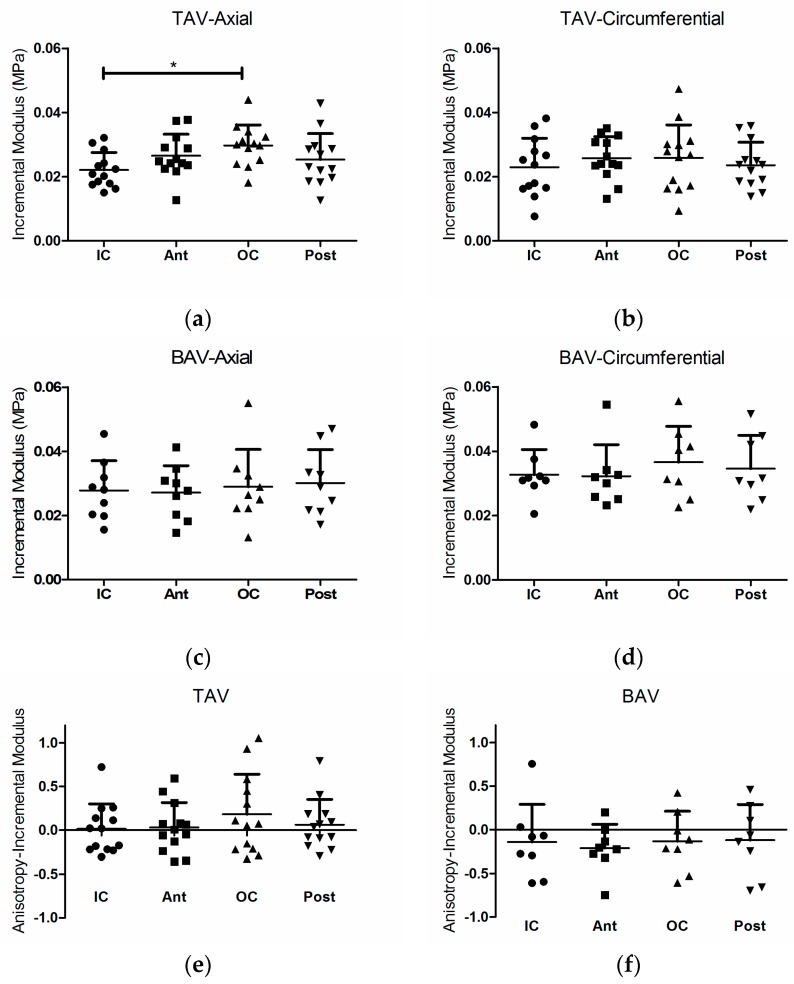
Variation of incremental modulus: (**a**) axial and (**b**) circumferential directions of the TAV group, and (**c**) axial and (**d**) circumferential directions of the BAV group, and anisotropy of the incremental modulus for the (**e**) TAV and (**f**) BAV groups. Only the axial modulus of the TAV group shows dependency on the region (*p* = 0.0305) (one-way ANOVA with repeated measure and Bonferroni’s multiple comparisons post-test). Asterisk denotes significant difference: * *p* < 0.05.

**Figure A2:**
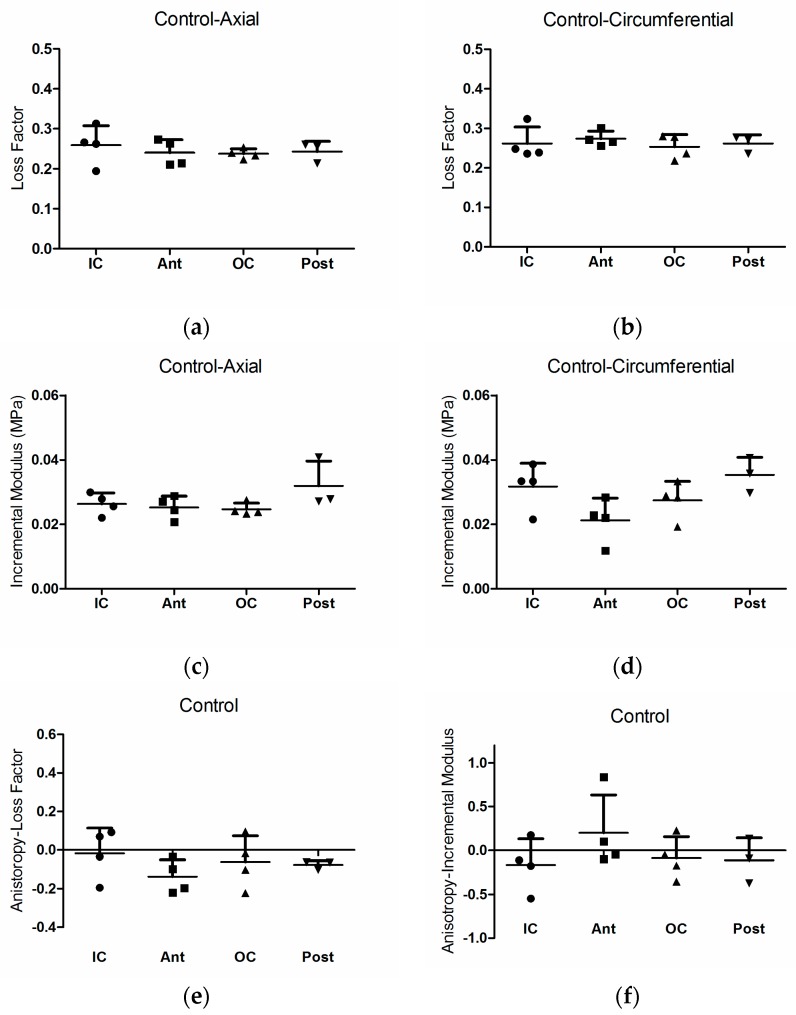
Figure presents data for all available control tissue. (**a**) Axial and (**b**) circumferential loss factor; (**c**) axial and (**d**) circumferential incremental modulus; and (**e**) loss factor anisotropy and (**f**) incremental modulus anisotropy for the control group (the mean values and standards deviations are shown).

For comparison purposes, the regional incremental modulus for the axial and circumferential directions and anisotropy of the incremental modulus for the BAV and TAV groups are presented in Appendix A, See Figure A1. Unlike the loss factor, the axial incremental modulus of the TAV group showed dependency on quadrants (one-way ANOVA with repeated measure, *p* = 0.0305). Figure A2 presents data for the incremental modulus and loss factor of all available control samples.

### 3.2. Structural Fiber Contents

**Figure 4:**
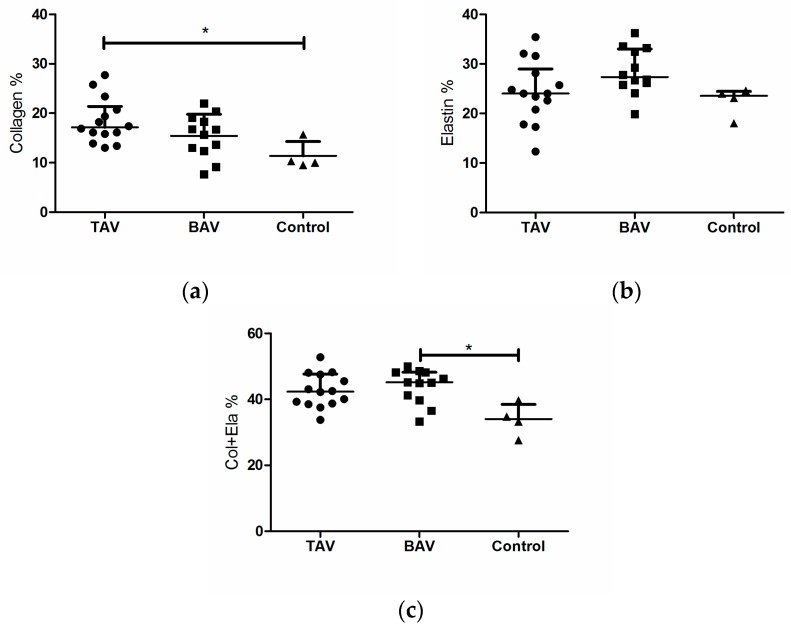

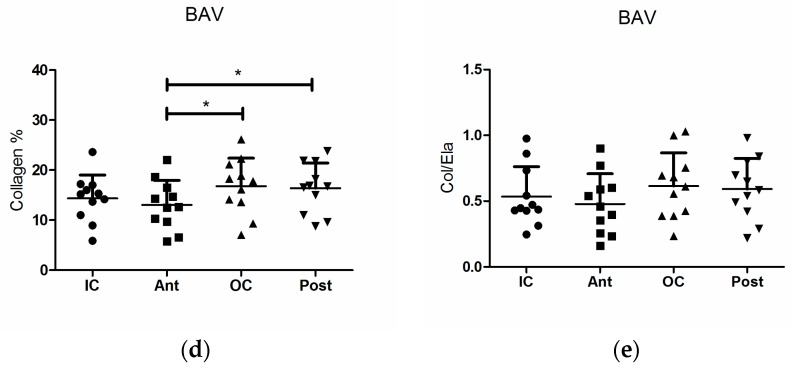
(**a**) Average collagen is dependent on groups (*p* = 0.0257); (**b**) Average elastin is dependent on groups (*p* = 0.0271); (**c**) The average content of collagen and elastin varies amongst the groups (*p* = 0.0260) (Kruskal-Wallis and Dunn’s multiple comparisons post-test); the images depict the median and interquartile. For the BAV group: (**d**) collagen depends on region (*p* = 0.0075); and (**e**) Col/Ela (*p* = 0.0458) (one-way ANOVA with repeated measure and Bonferroni’s multiple comparisons post-test). Asterisks denote significant difference: * *p* < 0.05.

Figure 4a shows the average collagen content of the quadrants significantly varies among tissue groups (Kruskal-Wallis, *p* = 0.0257). TAV tissue had the highest collagen content for which the median was significantly higher than the control group (Dunn’s multiple comparisons post-test, *p* < 0.05). The average elastin content and combined collagen + elastin content also varied between the groups (Kruskal-Wallis, *p* = 0.0271 and *p* = 0.0260, respectively, Figure 4b,c). BAV tissue had significantly more collagen + elastin than the control group (Dunn’s multiple comparisons post-test, *p* < 0.05). The TAV group showed similar increased collagen + elastin, but was not significantly different than control. There was no dependency between the collagen to elastin ratio (Col/Ela) and the tissue groups (Kruskal-Wallis, *p* = 0.0745, results are not shown).

Within the BAV group, collagen content varied with region (*n* = 11, one-way ANOVA with repeated measure, *p* = 0.0075, Figure 4d) with the OC and post regions containing more collagen than the anterior region (Bonferroni’s multiple comparisons post-test, *p* < 0.05). Figure 4e shows the variation of collagen to elastin ratio (Col/Ela) of the BAV group which was dependent on the location (one-way ANOVA with repeated measure, *p* = 0.0458). There was no significant regional variation in the elastin and collagen + elastin contents of the BAV groups (one-way ANOVA with repeated measure, *p* = 0.7590 and 0.1468, respectively). Additionally, the collagen content, collagen + elastin content and Col/Ela did not vary regionally in the TAV group (*n* = 14, one-way ANOVA with repeated measure, *p* = 0.3993, 0.4621, and 0.4835, respectively). Elastin varied regionally for the TAV group (one-way ANOVA with repeated measure, *p* = 0.0138) with the OC region containing more elastin than the Post region (one-way ANOVA with repeated measure, *p* < 0.05). Small tissue portions were sufficient for histology analysis, hence, the number of data available for the histology analysis were higher than the mechanical analysis.

### 3.3. Correlations

**Figure 5:**
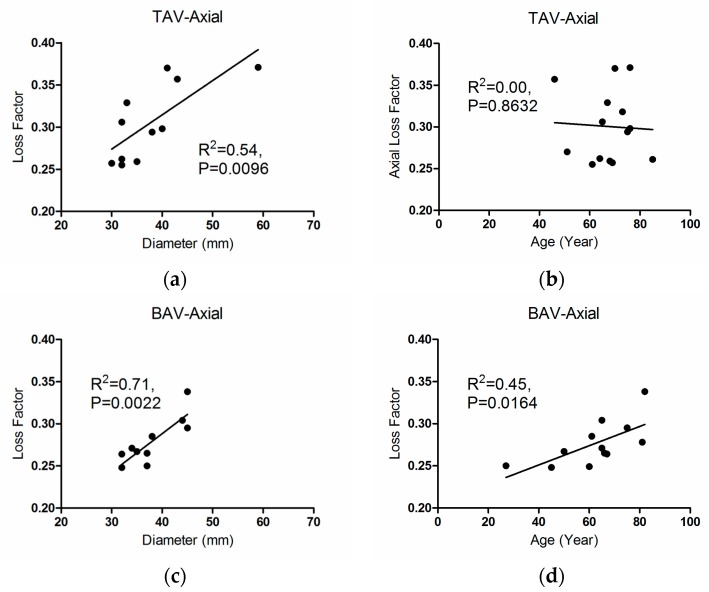
Average axial loss factor with respect to (**a**) aortic diameter for the TAV group; (**b**) age for the TAV group; (**c**) aortic diameter for the BAV group; and (**d**) age for the BAV group.

**Figure A3:**
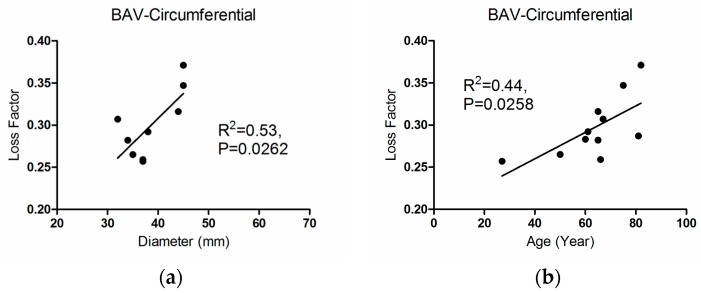
Average circumferential loss factor of the BAV group with respect to (**a**) diameter; and (**b**) age.

Figure 5 shows the correlations for axial loss factor with respect to patients’ age and diameter of both tissue groups. For the TAV group, axial loss factor correlated well with aortic diameter (*R*^2^ = 0.54, *p* = 0.0096, Figure 5a). There were significant correlations between axial and circumferential loss factor with aortic diameter for the BAV group (*R*^2^ = 0.71, *p* = 0.0022, Figure 5c, and *R*^2^ = 0.53, *p* = 0.0262, Figure A3a, respectively, for the axial and circumferential directions). Interestingly, we found meaningful correlations between loss factor and patients’ age for the BAV group (*R*^2^ = 0.45, *p* = 0.0146, Figure 5d, and *R*^2^ = 0.44, *p* = 0.0258, Figure A3b, respectively, in the axial and circumferential directions). These properties did not correlate for the TAV group. 

**Figure 6:**
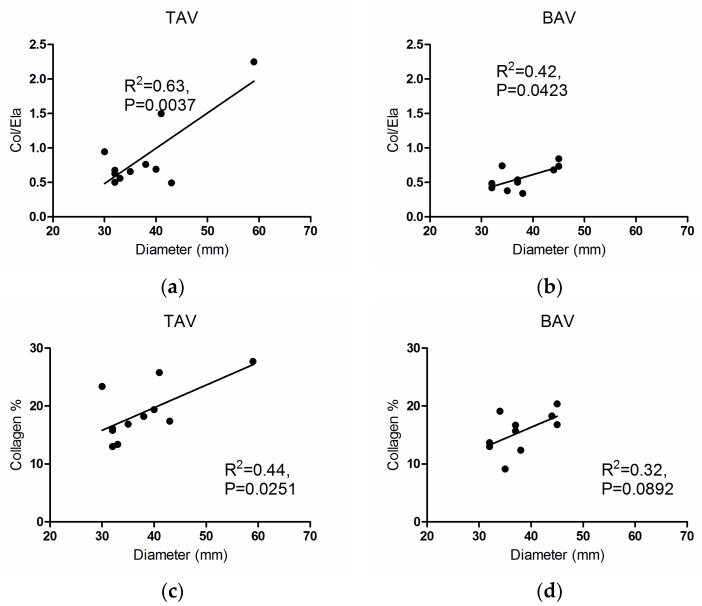
Col/Ela with respect to diameter for (**a**) the TAV; and (**b**) the BAV groups; collagen with respect to diameter for (**c**) the TAV; and (**d**) the BAV groups.

**Figure 7:**
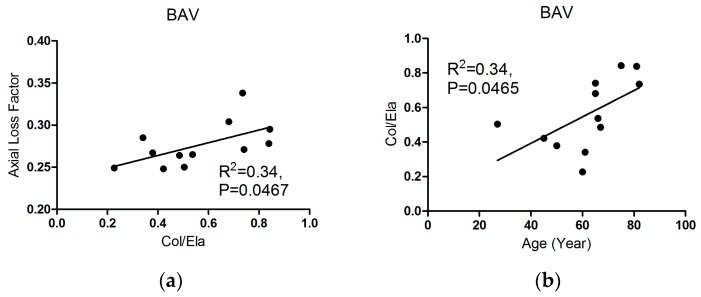
(**a**) Axial loss factor with respect to Col/Ela for the BAV group; (**b**) Col/Ela with respect to age for this group.

The variations of collagen content and Col/Ela with aortic diameter are compared in Figure 6. Col/Ela increased significantly with aortic diameter for both TAV and BAV groups (*R*^2^ = 0.63, *p* = 0.0037 and *R*^2^ = 0.42, *p* = 0.0423, respectively, Figure 6a,b). Unlike the BAV group, the collagen and diameter correlated positively in the TAV group (*R*^2^ = 0.44, *p* = 0.0251, Figure 6c,d). Figure 7a compares the variation of axial loss factor with respect to Col/Ela of the BAV group (*R*^2^ = 0.34, *p* = 0.0467). This figure also shows the significant increase of Col/Ela with age in this group (*R*^2^ = 0.34, *p* = 0.0465, Figure 7b). There were no meaningful correlations between the histological parameters and age for the TAV groups. 

**Figure 8:**
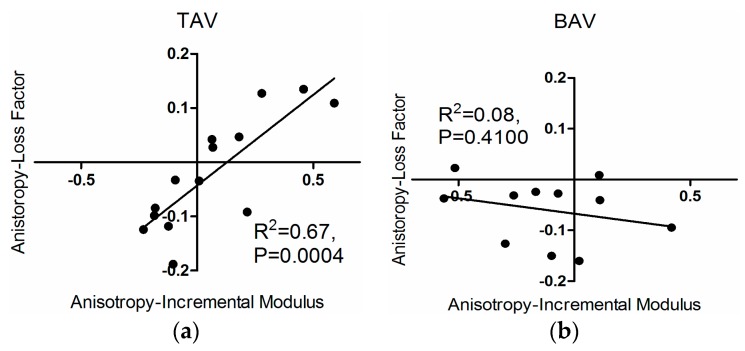
Loss factor anisotropy with respect to incremental modulus anisotropy for (**a**) the TAV; and (**b**) the BAV group.

We also found correlations between loss factor anisotropy and incremental modulus anisotropy for the TAV group (*R*^2^ = 0.67, *p* = 0.0004); a similar relationship was not observed for the BAV group (Figure 8).

## 4. Discussion

In this study, we examined the strip-biaxial loss factor, incremental modulus, and histological properties of aneurysmal and control ATAs. We compared the mechanical and histological properties with respect to valve type (BAV or TAV) and regional variation within each tissue sample. The results show that the anisotropy of the loss factor differs between TAV and BAV tissue. The loss factor may be a more robust mechanical metric in identifying regional remodelling of the aneurysmal tissue. 

The strip-biaxial experimental method was selected to determine the loss factor property of the tissue and to investigate the differences of the loss factor of for the axial and circumferential directions, systematically. While Chung et al. [15] used equi-biaxial tensile tests for examining tissue loss factor, in this work, the tissue samples were subjected to two sets of strip-biaxial tensile tests in the axial and circumferential directions. The strip-biaxial tensile tests help separate the response of the two directions and are useful for modelling purposes. This method determined that the loss factor resulted from the tissue strain energy due to strain changes in only one direction, since the immobile axis with zero strain did not contribute to the strain tissue energy level.

The study of loss factor anisotropy revealed important information on regional isotropy and differences between the tissue groups, although the axial and circumferential loss factor of the BAV and TAV tissue groups looked very much the same at the first glance (Figure 3). The loss factor was isotropic for the four quadrants of the TAV group whereas, for the BAV group, this factor behaved isotopically only for the OC and ant regions (ATAAs mainly rupture in these quadrants [27]). The loss factor anisotropic index was clearly negative in the IC and post regions of the BAV group, describing that the axial loss factor is smaller than the circumferential one in these regions. It appears that tissue remodelling could cause isotropic mechanical properties during the progression of the aneurysm. Another interesting fact is that loss factor and incremental modulus anisotropy co-vary in the TAV group but not in the BAV group. This could be due to increased collagen content in the TAV. The differences of regional anisotropy amongst the tissue groups are due to dissimilarities between the hemodynamics and remodelling processes of these groups. 

In general, the histological characteristics of the TAV tissue group in comparison to the BAV group show increased levels of collagen content, higher elastin loss, more fragmented elastin fibers, and less aligned elastin and collagen fibers (see Figure 2) [28,29]. As discussed by Collins et al., ATAAs associated with BAV show less changes in elastin fragmentation and loss, yet the tissue exhibit abnormal laminar structure where the elastic laminae collapsed on top of each other [28]. These findings can demonstrate the differences between the directional mechanical properties (anisotropy) and histological characteristics of the BAV and TAV tissue groups found in this study. Given the collective mechanical and histological data, we hypothesize that tissue remodelling affects the axial loss factor due to collapse of elastic fibers and their connection through the extracellular matrix (ECM). The results describe the importance of exploring the tissue behavior in the axial direction for improving the current clinical criterion, the aortic diameter [1]. 

We found that the axial and circumferential in vitro loss factors of aneurysmal aortas were significantly higher than those of control tissue (Mann-Whitney two *t*-test *p* = 0.0303 and 0.0341, respectively; the results are not shown). Similar results were reported in a study by Chung et al. [15]. Sokolis et al. also observed greater viscosity of ATAAs comparing to the control tissue through measuring the creep opening angle of the control and aneurysmal ATAs [22]. Similar to the axial loss factor obtained in this study, they also found higher fractional energy loss of TAV-ATAAs than control tissue (for all aortic quadrants). They observed greater energy loss in the axial direction for TAV-ATAAs in comparison to the circumferential direction. Unlike this study, Sokolis et al. reported anisotropy of the fractional energy loss for TAV-ATAAs. 

The interaction of blood flow and the ATAA in the physiological condition causes axial and circumferential stresses and strains fields in the aortic wall. Both directions work together and contribute to the energy absorption, redistribution, and dissipation. The energy of hysteresis loops dissipates through heat and can cause thermal stresses in the wall. This energy may cause the growth of micro defects in the pathological aortic wall and can harmfully affect the diseased tissue regeneration and ultimately result in its rupture (fatigue failure). The remodelling and collapse of elastin fibers, the orientation-dependent variation of scleroproteins [23], the direction of tears in ATAAs wall (mainly transversal), and the increase of ATAAs loss factor highlight the importance of the axial wall biomechanics. Exploring the loss factor with respect to morphology and orientations of scleroproteins, the content of new to mature fibers, and the role of extracellular matrix is an open area for future investigations. A limitation of this study was obtaining the loss factor from in vitro strip-biaxial tensile cycles, omitting the complex effect of surrounding tissue and the interaction of the blood flow and the aortic wall (fluid-structure interaction) of the in vivo condition. This study is also limited due to the small size of control group, measuring the stress and strain data using the sutures locations, the mixed orientation of the histological slides, and the potential effect of smooth muscle cells activations. A larger control tissue group, employing optical methods to measure the tissue strain [30], performing tensile tests by controlling the strain level of the central-region of the specimens, and activating the SMCs could provide more information on ATAAs rupture. Exploring the axial (longitudinal) and circumferential loss factor of the tissue in vivo can clarify further aspects of ATAAs biomechanics. The loss factor could provide a tangible patient-specific surrogate measure for clinical applications representing the mechanical properties of the aortic wall. This measure may be obtained in vivo non-invasively by employing imaging techniques, to obtain the aortic strain, and by using imperial or other advanced biomechanical methods, such as finite element analysis of blood flow and aortic wall interaction, to compute the dynamic stress exerted on the aortic wall during a heartbeat [31,32]. Our group is currently working on examining the in vivo loss factor.

## 5. Conclusions

The results indicate that the axial loss factor varies significantly amongst the three tissue groups (control, TAV and BAV). Likewise, significant differences were observed between the histological properties of the tissue groups. While loss factor is isotropic in all regions of the TAV group, it is on average only isotropic in the OC and ant quadrants of the BAV group. The differential pathology between TAV and BAV groups might suggest that the growth of the aneurysm may be a result of localized hemodynamic-driven remodelling of the wall for BAV. 

The correlations between the loss factor and aortic diameter were strong for both the BAV and TAV groups. Similarly, Col/Ela and diameter correlate in both groups. Additionally, the loss factor of the BAV group correlates with patients' age, and correlations between Col/Ela and age in the BAV group is significant. The results suggest that loss factor, which may be obtained non-invasively using medical imaging techniques, could be beneficial to describe the histopathology of aneurysmal tissue. This concept appears to be a powerful method to understand the biomechanics of aortic aneurysm progression from an energy perspective. Additionally, the axial characteristics of the aorta seem to play a key role in the biomechanics of ATAAs. 

## Figures and Tables

**Table 1:** The characteristics of patients’ population including control aortas (*n* = 4) and aneurysmal aortas categorized into the bicuspid (BAV) (*n* = 12) and tricuspid aortic valves (TAV) (*n* = 14) tissue groups.

Sample Number	Gender	Age (Year)	Aortic Diameter (mm)	Valve Type
1	M	65	44	BAV
2	F	82	45	BAV
3	M	61	38	BAV
4	F	66	37	BAV
5	F	27	37	BAV
6	M	50	35	BAV
7	M	67	32	BAV
8	M	75	45	BAV
9	M	65	34	BAV
10	M	45	32	BAV
11	M	81	48 *	BAV
12	M	60	50 *	BAV
13	M	51	58 *	TAV
14	F	70	41	TAV
15	M	61	32	TAV
16	M	64	32	TAV
17	M	76	59	TAV
18	M	65	32	TAV
19	M	75	38	TAV
20	M	73	38 *	TAV
21	M	67	33	TAV
22	M	69	30	TAV
23	M	76	40	TAV
24	M	68	35	TAV
25	M	46	43	TAV
26	F	85	50 *	TAV
27	M	49	37	Control
28	M	81	37	Control
29	M	52	24	Control
30	M	48	24	Control

Asterisks (*) denotes in vivo diameter.
